# Regulation of the Wnt/β-Catenin Signaling Pathway by Non-Coding RNAs in Esophageal Squamous Cell Carcinoma: Mechanisms, Translational Relevance, and Therapeutic Implications

**DOI:** 10.32604/or.2026.081222

**Published:** 2026-08-13

**Authors:** Chao Han, Ming Hou, Ruifeng Yang, Xiaoping Wei, Cheng Wang

**Affiliations:** Department of Thoracic Surgery, The Second Hospital & Second Clinical Medical College, Lanzhou University, Lanzhou, China

**Keywords:** Esophageal squamous cell carcinoma (ESCC), Wnt/β-catenin signaling pathway, non-coding RNA, epithelial-mesenchymal transition (EMT), chemoresistance

## Abstract

Esophageal Squamous Cell Carcinoma (ESCC) is a highly aggressive malignancy characterized by a poor long-term prognosis. Aberrant activation of the canonical Wnt/β-catenin pathway serves as a central oncogenic driver in ESCC, with large-scale genomic analyses revealing that most patients harbor alterations in pathway-associated genes. This signaling axis orchestrates a wide array of malignant phenotypes, including tumor proliferation, invasion, epithelial-mesenchymal transition (EMT), cancer stemness, and therapeutic resistance. Therefore, this review aims to provide a comprehensive synthesis of the multifaceted crosstalk between various ncRNA classes and the Wnt/β-catenin axis, highlighting their roles in ESCC progression and their potential as mechanism-based clinical tools. Emerging evidence identifies non-coding RNAs (ncRNAs)—including microRNAs (miRNAs), long non-coding RNAs (lncRNAs), and circular RNAs (circRNAs)—as key regulators of this pathway in ESCC. In this article, we systematically summarize the molecular mechanisms by which 36 unique ncRNA species (20 miRNAs, 10 lncRNAs, and 6 circRNAs) modulate Wnt signaling. Oncogenic ncRNAs promote malignant progression by suppressing negative regulators within complex competing endogenous RNA (ceRNA) networks, whereas tumor-suppressive ncRNAs inhibit pathway activation by targeting Wnt ligands, receptors, or downstream effectors. Furthermore, we discuss the translational relevance of ncRNA-based biomarkers for risk stratification and prognosis in clinical cohorts. Finally, we evaluate pharmacological strategies for ncRNA-based therapeutics while highlighting critical challenges related to delivery efficiency, specificity, molecular stability, off-target effects, tumor heterogeneity, and clinical validation. Ultimately, a clearer understanding of the ncRNA-Wnt regulatory network is essential for developing personalized, mechanism-based diagnostic and therapeutic strategies.

## 1 Introduction

Esophageal cancer (EC) remains a major global health burden and ranks among the leading causes of cancer-related mortality worldwide. According to recent epidemiological data, EC is the seventh most common cause of cancer-related death globally. It is characterized by particularly high incidence and mortality rates in China, which accounts for more than half of all newly diagnosed cases and deaths globally [1,2,3,4,5]. Histologically, EC is primarily classified into esophageal squamous cell carcinoma (ESCC) and esophageal adenocarcinoma (EAC), which differ markedly in their geographic distribution, etiology, and molecular characteristics. ESCC predominates in East Asia and developing regions, whereas EAC is more common in Western countries [2]. Despite advances in diagnostic techniques and multimodal therapy, the prognosis for ESCC continues to be dismal. According to the 2022 GLOBOCAN report, the high mortality is evidenced by approximately 445,129 deaths out of 510,716 new cases globally, with long-term survival remaining significantly limited. This unfavorable outcome is largely attributed to the fact that many patients are still diagnosed at intermediate or advanced stages, which often precludes them from receiving optimal surgical treatment [6,7]. Late-stage diagnosis, aggressive tumor behavior, early metastasis, and frequent resistance to chemoradiotherapy collectively contribute to these poor clinical outcomes. Consequently, there is an urgent need to elucidate the molecular mechanisms underlying ESCC progression and therapeutic resistance in order to identify novel biomarkers and actionable therapeutic targets.

Among the primary oncogenic signaling pathways implicated in ESCC, the canonical Wnt/β-catenin pathway plays a central role in governing cell proliferation, differentiation, stemness, and tissue homeostasis [8,9]. Aberrant activation of this pathway is documented in a substantial proportion of ESCC cases, with genomic analyses revealing that up to 91% of patients harbor alterations in Wnt/β-catenin pathway-associated genes [10]. Dysregulated Wnt signaling promotes tumor initiation, invasion, metastasis, epithelial-mesenchymal transition (EMT), and resistance to chemoradiotherapy, serving as a critical driver of ESCC malignancy [10,11].

In recent years, non-coding RNAs (ncRNAs)—including microRNAs (miRNAs), long non-coding RNAs (lncRNAs), and circular RNAs (circRNAs)—have emerged as critical modulators of cancer-associated signaling networks. miRNAs are endogenous small RNAs (18–24 nucleotides) that suppress gene expression mainly by binding to the 3′untranslated region (3′-UTR) of target mRNAs [12]. lncRNAs are ncRNAs longer than 200 nucleotides that often regulate target gene expression through ceRNA mechanisms by sponging specific miRNAs, thereby influencing key biological processes such as proliferation, survival, and apoptosis [13]. circRNAs are covalently closed circular ncRNAs with high resistance to nuclease degradation, conferring them with exceptional stability and enabling them to form complex regulatory networks in ESCC [14]. These molecules modulate gene expression at the transcriptional, post-transcriptional, and epigenetic levels [15,16]. In the context of esophageal cancer, ncRNAs are increasingly recognized as key regulators of tumor progression and as potential biomarkers or therapeutic targets [12,17,18]. Notably, accumulating evidence demonstrates that ncRNAs can modulate Wnt/β-catenin signaling through multiple mechanisms, including the direct targeting of pathway components, the regulation of upstream modulators, and participation in competing endogenous RNA (ceRNA) networks that fine-tune gene expression [12,15]. Aberrant activation of the Wnt/β-catenin signaling pathway, driven by various genetic and epigenetic alterations, is a fundamental hallmark of ESCC and is closely associated with poor clinical outcomes [19].

Although previous reviews have discussed non-coding RNAs in esophageal cancer or Wnt signaling in cancer more broadly, an updated synthesis specifically focusing on how different ncRNA classes converge on the Wnt/β-catenin network in ESCC remains to be established [9,17,18,20]. Recent advancements have underscored the multifaceted roles of ncRNAs in the diagnosis, progression, and prognosis of esophageal cancer, offering new dimensions for understanding its molecular complexity [21,22,23]. However, despite these insights, the integrated regulation of the Wnt/β-catenin signaling axis by diverse ncRNA species awaits full elucidation, particularly regarding the impact of RNA modifications on transcript stability [24]. In particular, the field still lacks a unified framework linking direct miRNA-mediated repression, lncRNA/circRNA-associated ceRNA regulation, phenotype-specific outputs, and translational potential.

Therefore, this review aims to elucidate the mechanistic landscape of how miRNAs, lncRNAs, and circRNAs regulate the Wnt/β-catenin signaling pathway in ESCC. We further discuss the translational implications of these regulatory networks pertaining to biomarker development, therapeutic resistance, and ncRNA-based intervention strategies, while underscoring current limitations and future research priorities.

## 2 Overview of the Wnt Signaling Pathway

**Figure 1:**
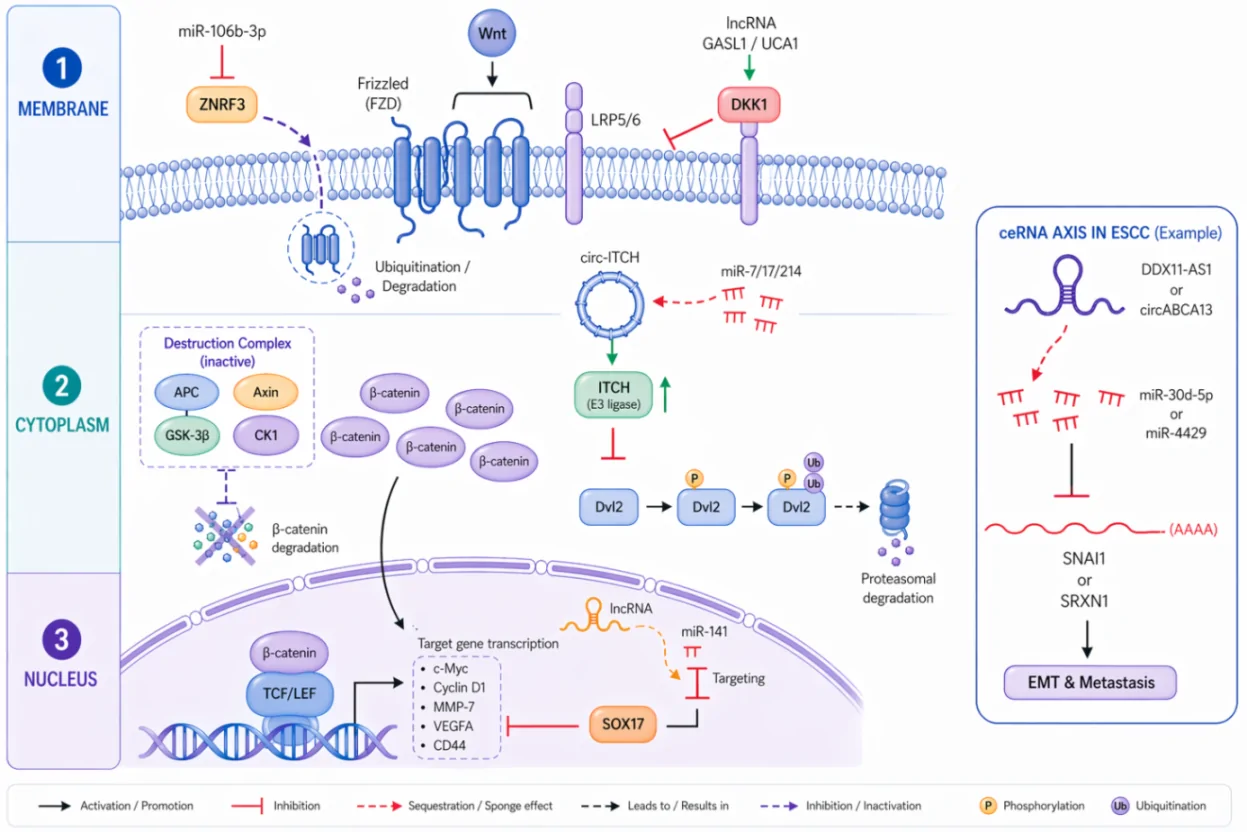
**Comprehensive regulatory network of the Wnt/β-catenin signaling pathway modulated by ncRNAs in esophageal squamous cell carcinoma (ESCC).** The canonical pathway is orchestrated by a multi-layered ncRNA network spanning the membrane, cytoplasm, and nucleus. (1) At the membrane level, oncogenic miR-106b-3p maintains receptor stability by inhibiting the E3 ligase ZNRF3, while tumor-suppressive lncRNAs (GASL1, UCA1) block signaling by upregulating the antagonist DKK1. (2) In the cytoplasm, the circ-ITCH (circBase ID: hsa_circ_0001141)/ITCH axis facilitates the ubiquitination and proteasomal degradation of Dvl2, thereby reinforcing the destruction complex (comprising APC, Axin, GSK-3β, and CK1) to promote β-catenin turnover. (3) Within the nucleus, miR-141 lifts the inhibitory constraint of SOX17, leading to β-catenin/TCF/LEF-mediated transcription of genes driving proliferation (c-Myc, Cyclin D1), metastasis (MMP-7, CD44), and angiogenesis (VEGFA). (Inset) Specific ceRNA axes in ESCC, such as DDX11-AS1/miR-30d-5p and/miR-4429, sequester miRNAs to activate downstream EMT effectors (SNAI1, SRXN1), fueling malignant progression.

The Wnt gene was originally discovered at the integration site of Int-1 in mouse mammary tumors and is homologous to the wingless gene in Drosophila. Due to the functional similarities between these proteins, the terms were fused to form the “Wnt” family [8,25]. Wnt signaling is broadly classified into canonical and non-canonical pathways. The canonical Wnt pathway, also known as the Wnt/β-catenin pathway (Fig. 1), relies on the stabilization and activation of β-catenin, its nuclear translocation, and the subsequent activation of target genes mediated by transcription factors including T-cell factor/lymphoid enhancer factor (TCF/LEF).

This pathway is particularly critical in ESCC, where it functions as a primary driver of malignant transformation [9,10,19]. In contrast, the non-canonical Wnt pathways primarily encompass the planar cell polarity (PCP) pathway, the receptor tyrosine kinase (RTK) pathway, and the Ca^2^^+^ pathway [8,26]. The PCP pathway activates c-Jun N-terminal kinase (JNK), which in turn stimulates the activator protein-1 (AP-1) complex; the RTK pathway involves the activation of receptor tyrosine kinases; and the Ca^2^^+^ pathway enhances intracellular Ca^2^^+^ levels to activate protein kinase C (PKC), consequently affecting cell adhesion and intracellular signaling. Previous studies suggest that esophageal cancer development is primarily associated with the canonical Wnt pathway.

In the absence of Wnt ligand stimulation, β-catenin is continuously degraded by a multiprotein “destruction complex” comprising Axin, adenomatous polyposis coli (APC), glycogen synthase kinase-3β (GSK-3β), casein kinase I (CK1), Wilms tumor gene on the X chromosome (WTX) [27], protein phosphatase 2A (PP2A), and β-transducin repeat-containing protein (β-TrCP) [28]. Within this complex, CK1α initially phosphorylates β-catenin at Ser45, serving to prime the protein for sequential phosphorylation by GSK-3β at Thr41, Ser37, and Ser33. These modifications generate a β-TrCP recognition motif, triggering ubiquitination and subsequent proteasomal degradation. Axin functions as a molecular scaffold that facilitates the assembly of these kinases and β-catenin, effectively maintaining low cytoplasmic β-catenin levels [29,30].

Moreover, in the absence of Wnt ligands, the transcriptional corepressors Groucho (Gro) in invertebrates and transducin-like enhancer of split (TLE) in vertebrates compete with β-catenin for binding to TCF/LEF, thus repressing the transcription of Wnt target gene [31]. Groucho can also recruit histone deacetylases (HDACs) to promote histone deacetylation and chromatin condensation, further suppressing transcription [32]. To date, 19 distinct Wnt ligands have been identified, including Wnt3a, Wnt1, and Wnt5a. These ligands are secreted glycoproteins that undergo extensive palmitoylation and glycosylation [33].

Upon ligand stimulation, Wnt binds to the seven-transmembrane Frizzled (FZD) receptors [34], forming a ternary membrane complex with the co-receptors low-density lipoprotein receptor-related protein 5/6 (LRP5/6). This triggers the sequential phosphorylation of LRP5/6 by GSK-3β and CK1, facilitating the recruitment of AXIN1/2 to the plasma membrane. Subsequently, Dishevelled (DVL) is recruited via its DIX domain through cooperative anchoring involving the intracellular tail of FZD and the LRP5/6-AXIN complex. The interaction between DVL and AXIN disrupts the destruction complex, subsequently inhibiting β-catenin phosphorylation and ubiquitination [35].

Stabilized cytoplasmic β-catenin translocates into the nucleus to form a functional complex with TCF/LEF, where it recruits coactivators such as CBP/p300 to initiate the transcription of Wnt target genes [36]. These activated target genes include key regulators of cell cycle progression (e.g., c-myc, N-myc, c-jun, cyclin D1, Sox9) [37], as well as factors promoting metastatic dissemination (e.g., MMP-7, VEGF) [9]. Given the robust association between aberrant Wnt/β-catenin activation and tumorigenesis, elucidating its mechanistic role in ESCC is pivotal for identifying novel anticancer targets and developing effective therapeutic strategies. The clinical significance of this pathway in ESCC is further reinforced by large-scale genomic studies. Analysis of the TCGA-ESCC cohort highlights a high frequency of dysregulation in core pathway components, like CTNNB1 mutations, AXIN2 overexpression, and the constitutive activation of FZD receptors. These genomic landscapes underscore the Wnt/β-catenin axis as a primary driver of esophageal oncogenesis and a potent predictor of patient prognosis [19].

## 3 Regulation of Wnt/β-Catenin Signaling by ncRNAs in ESCC

Non-coding RNAs (ncRNAs), including microRNAs (miRNAs), long non-coding RNAs (lncRNAs), and circular RNAs (circRNAs), are pivotal regulators of gene expression and cancer progression [15,16]. A growing body of evidence indicates that these ncRNAs play central roles in modulating the Wnt/β-catenin pathway, a key signaling axis governing malignant transformation. In ESCC, ncRNAs regulate critical components of this pathway, such as β-catenin (the primary nuclear effector), APC (a core member of the destruction complex), and DVL (an upstream transducer), effectively influencing multiple aspects of tumor progression. 

Specifically, miRNAs in ESCC profoundly shape malignant phenotypes—including tumor growth, invasion, metastasis, radiosensitivity, and EMT—by targeting key components of Wnt/β-catenin signaling. Similarly, aberrantly expressed lncRNAs in ESCC function as either oncogenes or tumor suppressors, contributing to tumor progression through the direct or indirect regulation of the Wnt/β-catenin axis. Analogous to lncRNAs, circRNAs often function as “molecular sponges” to sequester miRNAs, hence alleviating their inhibitory effects on downstream mRNA targets and modulating Wnt/β-catenin signaling, which is a recognized driver of ESCC initiation and progression [38,39]. Oncogenic circRNAs are frequently upregulated in ESCC and promote malignant phenotypes through this competitive sponge mechanism. To facilitate a more integrated understanding, the following sections are organized thematically according to the functional impacts of these ncRNAs on ESCC, focusing on proliferation and cell-cycle progression, epithelial-mesenchymal transition (EMT), invasion and metastasis, and chemoradiotherapy resistance, rather than discussing each ncRNAs class in isolation.

### 3.1 Promotion of Cell Proliferation and Cell Cycle Progression

One primary mechanism by which ncRNAs promote ESCC progression is through the sustained activation of Wnt/β-catenin signaling, which drives cell proliferation and cell-cycle progression. Oncogenic miRNAs are frequently overexpressed in ESCC and often enhance Wnt signaling by suppressing endogenous pathway inhibitors. A notable example is miR-141, which is markedly upregulated in ESCC and directly targets SOX17 [21]. As a member of the SRY-related HMG-box transcription factor family, SOX17 antagonizes the TCF/LEF complex and suppresses Wnt signaling by promoting the β-catenin/TCF degradation through a GSK-3β-independent mechanism [19]. Consequently, the miR-141-mediated downregulation of SOX17 leads to aberrant Wnt/β-catenin activation and accelerates tumor cell proliferation [21]. In addition, hypermethylation of the SOX17 promoter may cooperate with miR-141 to further facilitate tumorigenesis. 

Similarly, miR-106b-3p promotes Wnt signaling by targeting ZNRF3, an E3 ubiquitin ligase that negatively regulates the pathway by mediating the K48-linked polyubiquitination and proteasomal degradation of Wnt receptors (FZD/LRP6) [24]. While DKK1 is traditionally recognized as a potent antagonist of the canonical Wnt pathway via its binding to LRP5/6 receptors [40], its role in ESCC exhibits significant complexity and context-dependency. Emerging evidence suggests that DKK1 may also be involved in compensatory feedback loops or exhibit non-canonical functions. For instance, it has been observed that a reduction in miR-33a-5p leads to abnormally high DKK1 levels, which, paradoxically, promote malignant progression through non-canonical pathways such as PI3K/AKT [41]. In this context, DKK1 inhibition has been shown to actually reduce overall oncogenic activity, highlighting a functional shift from its classical inhibitory role. Such paradoxical findings underscore the necessity for future research to account for the heterogeneous genetic backgrounds and crosstalk with other signaling cascades when evaluating ncRNA-Wnt interactions in ESCC.

Other miRNAs also regulate proliferative signaling through distinct Wnt-related targets. miR-30a-3p is markedly downregulated in ESCC and suppresses cell proliferation by targeting WNT2 [42]. Additional tumor-suppressive miRNAs influence proliferation, invasion, and angiogenesis through Wnt-associated targets. For instance, miR-92a-2-5p is downregulated and inhibits proliferation by targeting PRDX2, an oncogenic factor that promotes ESCC growth through Wnt activation [43,44]. Given that Wnt signaling is essential for angiogenesis [45]; SERPINH1 emerges as an upstream activator whose high expression promotes VEGFA production and angiogenesis. miR-29c-3p, which is also reduced in ESCC, targets SERPINH1 and suppresses Wnt/β-catenin activation, thereby inhibiting proliferation while simultaneously reducing VEGFA expression [46]. 

Collectively, miRNAs constitute a crucial regulatory layer of the Wnt/β-catenin pathway in ESCC. Oncogenic miRNAs (e.g., miR-141, miR-106b-3p, and miR-942) promote malignant progression by abrogating inhibitory constraints, whereas tumor-suppressive miRNAs (e.g., miR-1275, miR-30a-3p, miR-210, miR-10527-5p, miR-92a-2-5p, and miR-29c-3p) suppress pathway activation and counteract key malignant phenotypes. 

In addition to these microRNA-mediated mechanisms, long non-coding transcripts introduce an independent layer of regulation within the same pathway. For example, the lncRNA PVT1 promotes G2/M phase progression and suppresses apoptosis by positively regulating the Wnt axis [47]. Through these interconnected layers, together, these findings indicate that both miRNAs and lncRNAs contribute to uncontrolled ESCC growth by enhancing Wnt/β-catenin pathway activity or by neutralizing endogenous inhibitory constraints. Representative ncRNAs involved in cell proliferation and cell cycle progression are summarized in Table 1.

**Table 1:** Promotion of Cell Proliferation and Cell Cycle Progression.

Category	ncRNA/Axis	Expression in ESCC	Biological Effects	Clinical Relevance	Potential Therapeutic	Experimental Validation Level	Reference
miRNA	miR-141/SOX17	Upregulated	Promotes proliferation	Early detection marker; Linked to alcohol consumption	SOX17 demethylation or miR-141 inhibition	Clinical + *in vitro*	[21]
miRNA	miR-33a-5p/DKK1/PI3K/AKT	Downregulated	Suppresses proliferation, migration and invasion;	5-year OS (Overall Survival); TNM staging; Differentiation degree; Diagnostic marker	miR-33a-5p mimics or DKK1 inhibition	Clinical + *in vitro* + *in vivo*	[41]
miRNA	miR-30a-3p/WNT2/3′-UTR	Downregulated	Inhibits proliferation	Advanced progression; Poor prognosis; 5-year OS	miR-30a mimics or long-acting miR-30a	Clinical + *in vitro* + *in vivo*	[42]
miRNA	miR-92a-2-5p	Downregulated	Inhibits proliferation and invasion	TNM stage and Prognostic biomarker	miR-92a-2-5p inhibitors or PRDX2 inhibition	Clinical + *in vitro* + *in vivo*	[43]
miRNA	miR-29c-3p/SERPINH1	Downregulated	Suppresses proliferation, invasion and angiogenesis; promotes apoptosis	TNM stage and poor differentiation; Prognostic indicator	miR-29c-3p mimics or SERPINH1 inhibition	Clinical + *in vitro* + *in vivo*	[46]
lncRNA	PVT1/β-catenin, TCF-7, GSK-3β, Axin1	Upregulated	Promotes proliferation; Inhibits apoptosis; Accelerates G2/M transition	poor prognosis and OS; Diagnostic biomarker	Knockdown of PVT1	Clinical + *in vitro* + *in vivo*	[47]

### 3.2 Induction of EMT, Invasion, and Metastasis

Given the robust association between EMT and poor prognosis, identifying additional miRNAs that regulate EMT via Wnt signaling persists as a critical priority for the development of targeted therapeutic strategies. EMT represents a pivotal mechanistic link between Wnt pathway activation and metastasis, and is typically characterized by the loss of E-cadherin and concomitant increase N-cadherin and vimentin. Multiple downregulated miRNAs suppress EMT and tumor progression by targeting key Wnt pathway components [20]. For example, miR-210 is significantly downregulated in ESCC and inhibits Wnt/β-catenin signaling by targeting FBXO31, subsequently reducing the level of activated β-catenin, c-Myc, and cyclin D1, which in turn suppressing EMT and inhibiting invasion, migration, proliferation, and survival [48].

Tumor-suppressive ncRNAs, which are typically silenced in ESCC, function as critical brakes on Wnt/β-catenin signaling by targeting positive regulators such as Wnt ligands (Wnt1, Wnt2) or upstream activators. These miRNAs counteract malignant phenotypes, including EMT and radioresistance, by modulating the Wnt/β-catenin axis. Similarly, miR-10527-5p is significantly diminished in both plasma exosomes and tumor tissues of ESCC patients exhibiting lymph node metastasis. It suppresses Wnt/β-catenin signaling and EMT by directly targeting Rab10. Rab10 overexpression reverses these inhibitory effects and restores β-catenin activity [49].

While these microRNA species focus primarily on post-transcriptional antagonist targeting, long non-coding transcripts coordinate a complementary layer of regulation by directly interacting with core downstream components. lncRNAs also regulate core Wnt proteins (e.g., β-catenin) and downstream target genes (e.g., WISP1, VEGF-A, MMP2, ICAM-1), subsequently promoting tumor progression. The lncRNA CCAT2 is significantly upregulated in ESCC and correlates with advanced TNM stage, larger tumor size, and poor prognosis. CCAT2 reinforces the Wnt axis to drive metastatic progression; its depletion markedly reduces β-catenin and its pro-metastatic effector WISP1, ultimately attenuating tumor dissemination [50]. FEZF1-AS1 is associated with migration and invasion by upregulating β-catenin; its knockdown reduces β-catenin expression and metastatic potential [51]. Additionally, DUXAP8 activates β-catenin and its downstream targets cyclin D1 and c-Myc, establishing a self-sustaining oncogenic loop that fuels progression and metastasis [52].

Another major class of oncogenic lncRNAs functions primarily through ceRNA networks, indirectly regulating Wnt signaling and EMT. For instance, DDX11-AS1 is highly expressed in ESCC and is strongly associated with poor prognosis and advanced clinicopathological features [53]. It sponges miR-30d-5p, effectively alleviating the repression of the EMT transcription factors SNAI1 and ZEB2, which leads to E-cadherin suppression and EMT activation. This cascade facilitates the nuclear translocation of β-catenin and the subsequent activation of downstream targets such as c-Myc, MMP-7, CD44, and cyclin D1, ultimately enhancing proliferation, migration, and invasion [53,54]. Consistently, SNHG16 has been shown to activate the β-catenin signaling pathway, thus promoting the proliferative and inhibits the invasive capacity of ESCC cells [55]. These findings suggest that DDX11-AS1 and SNHG16 contribute to EMT and Wnt pathway dysregulation through distinct ceRNA axes, making them attractive targets for RNA-based intervention strategies.

Conversely, several tumor-suppressive lncRNAs are downregulated in ESCC and inhibit tumor progression by negatively regulating Wnt/β-catenin signaling. lncRNA-NEF is reduced in ESCC, and its overexpression decreases β-catenin protein levels while suppressing proliferation, migration, and invasion. Notably, NEF expression is independent of Wnt activation, yet its inhibitory efficiency is sensitive to pathway status, suggesting that NEF functions as a downstream gatekeeper [56]. LINC00675 is also downregulated and inhibits Wnt signaling by reducing the expression of β-catenin, cyclin D1, and c-Myc expression, while reversing EMT through the upregulation of the E-cadherin and the downregulation of N-cadherin and vimentin [57]. Furthermore, the lncRNAs GASL1 and UCA1 transcriptionally upregulate DKK1, consequently dampening Wnt3a/β-catenin nuclear accumulation [58,59].

Overall, lncRNAs exert dual regulatory roles in ESCC: oncogenic lncRNAs promote pathway activation and malignant phenotypes, whereas tumor-suppressive lncRNAs restrain pathway activity and EMT. circABCA13 (Circbase ID: hsa_circ_0001707) is significantly elevated in ESCC and correlates with advanced TNM stage and poor prognosis. Its overexpression promotes proliferation, migration, invasion, and EMT, while its knockdown reverses these phenotypes. Hsa_circ_0001707 sponges miR-4429 to upregulate SRXN1, which triggers the Wnt axis by facilitating β-catenin accumulation and the phosphorylation of GSK3β (Ser9). This drives upregulation of cyclins (Cyclin D1/D3) and EMT effectors (N-cadherin, MMP-2, MMP-9) while suppressing E-cadherin, accelerating ESCC progression [39].

In summary, the transition from an epithelial to a mesenchymal state in ESCC is orchestrated by a complex ncRNA network that converges on β-catenin stability and its subsequent nuclear activity. Representative ncRNAs involved EMT, Invasion, and Metastasis are summarized in Table 2.

**Table 2:** Induction of EMT, Invasion, and Metastasis.

Category	ncRNA/Axis	Expression in ESCC	Biological Effects	Clinical Relevance	Potential Therapeutic	Experimental Validation Level	Reference
miRNA	miR-210/FBXO31/β-catenin, c-Myc and cyclin D1; E-cadherin, Vimentin and N-cadherin	Downregulated	Inhibits proliferation, Suppresses EMT, migration and invasion	Predicts poor prognosis; Aggressive tumor progression; TNM stage	miR-210 restoration or FBXO31 inhibition	Clinical + *in vitro* + *in vivo*	[48]
miRNA	miR-10527-5p/Rab 10/β-catenin, pGSK3βSer9; E-cadherin, N-cadherin, Vimentin	Downregulated	Suppresses migration, invasion and EMT	Diagnostic biomarker	Exosome-based therapy target	Clinical + *in vitro* + *in vivo*	[49]
lncRNA	CCAT2/β-catenin/WISP1	Upregulated	Promotes proliferation and invasion	TNM stage, and poor overall survival	Silencing lncRNA CCAT2	Clinical + *in vitro* + *in vivo*	[50]
lncRNA	FEZF1-AS1/β-catenin/Wnt signaling	Upregulated	Promotes migration and invasion	Potential biomarker; TNM stage	FEZF1-AS1 knockdown	Clinical + *in vitro*	[51]
lncRNA	DUXAP8/β-catenin/c-Myc, Cyclin D1	Upregulated	Promotes proliferation, invasion and EMT	TNM stage and poor prognosis	Silencing DUXAP8	Clinical + *in vitro*	[52]
lncRNA	DDX11-AS1/miR-30d-5p/SNAI1, ZEB2; β-catenin/c-Myc, MMP-7, CD44, cyclin D1	Upregulated	Promotes EMT, proliferation, migration and invasion	TNM stage and poor prognosis	DDX11-AS1 knockdown or miR-30d-5p restoration	Clinical + *in vitro*	[53]
lncRNA	SNHG16/Wnt/β-catenin/c-Myc, β-catenin, cyclin D1	Upregulated	Promotes proliferation, invasion, EMT, migration and invasion; inhibits apoptosis	TNM stage and poor prognosis	Silencing SNHG16	Clinical + *in vitro*	[55]
lncRNA	lncRNA-NEF/β-catenin	Downregulated	Suppresses proliferation, migration and invasion	TNM stage and poor prognosis; Diagnostic biomarker	Overexpression of NEF	Clinical + *in vitro*	[56]
lncRNA	LINC00675/β-catenin/c-Myc, Cyclin D1; E-cadherin, N-cadherin, Vimentin	Downregulated	Suppresses proliferation; promotes apoptosis, invasion and EMT	TNM stage and poor prognosis	Overexpression of LINC00675	Clinical + *in vitro* + *in vivo*	[57]
lncRNA	GASL1/DKK1/Wnt3a/β-catenin/CDK2, CyclinD1, c-Myc	Downregulated	Suppresses proliferation, migration and invasion	Potential diagnostic biomarker; poor survival	Overexpression of GASL1	Clinical + *in vitro* + *in vivo*	[58]
lncRNA	UCA1/DKK1/β-catenin/Wnt signaling	Downregulated	Suppresses proliferation and migration	Potential diagnostic biomarker; ESCC risk	Overexpression of UCA1	Clinical + *in vitro*	[59]
circRNA	hsa_circ_0001707/miR4429/SRXN1/Wnt/β-catenin, GSK3β, N-cadherin/MMP-2, MMP-9; E-cadherin	Upregulated	Promotes proliferation migration, invasion and EMT	TNM stage; diagnostic biomarker; poor prognosis	hsa_circ_0001707 knockdown; miR-4429 restoration; SRXN1 inhibition	Clinical + *in vitro* + *in vivo*	[39]

### 3.3 Modulation of Chemoradiotherapy Resistance and Stemness

Beyond their mechanistic roles, miRNAs have profound clinical implications in modulating drug resistance in esophageal cancer. As highlighted by Yang et al. (2017) [60], specific miRNA signatures can serve as predictive biomarkers for therapeutic response, particularly in efforts to overcome chemoresistance to conventional agents [13]. While some ncRNAs primarily drive initial tumor growth, a distinct subset—such as miR-301a and miR-1275—serves as the molecular bridge between aberrant Wnt signaling and acquired therapeutic resistance. For instance, miR-942 overexpression targets three layers of Wnt pathway suppressors—sFRP4, GSK-3β, and TLE1—which induces the nuclear accumulation of β-catenin and promoting cancer stem-like traits. This expansion of the CD90^+^ subpopulation is evidenced by upregulation of pluripotency markers, including ABCG2, KLF4, SOX2, OCT4 and NANOG [61,62].

Parallel to their roles in growth regulation, miR-301a and miR-1275 are significantly depleted in radioresistant ESCC models, exemplified by KYSE-150R. Their downregulation relieves the inhibitory brake on the 3′-UTR of WNT1, consequently driving Wnt activation and establishing a radioresistance phenotype [63,64]. miR-1275 in particular, suppresses EMT marker switching by downregulating N-cadherin and vimentin while upregulating E-cadherin—an effect that can be reversed by WNT1 overexpression [63]. 

Distinct from these linear microRNA elements, circular RNA transcripts introduce an additional structural layer of radioresistance control through competitive sponging networks. In radioresistant KYSE-150R cells, circRNA_100367 (Circbase ID: hsa_circRNA_0014879) is markedly increased, where it functions as a molecular sponge for miR-217, This sequestration results in the upregulation of Wnt3 and subsequent elevation of β-catenin. Notably, Wnt3 knockdown reverses these effects, indicating that the hsa_circRNA_0014879 axis plays a central role in coordinating a multifaceted malignant program, including EMT-driven radioresistance [65].

Beyond radiotolerance, specialized circRNAs also contribute significantly to chemotherapy resistance. Cisplatin (DDP) is a standard first-line regimen for advanced ESCC; however, acquired resistance ultimately leads to treatment failure and represents a major cause of mortality [66,67,68,69]. Several circRNAs have been implicated in this process. For example, hsa_circ_0000277 is highly expressed in advanced ESCC and acts as a sponge for miR-873-5p, releasing the repression on SOX4 to trigger the Wnt axis. This cascade subsequently upregulates anti-apoptotic effectors Bcl-2 and Survivin, thus promoting tumor growth and conferring cisplatin resistance. Its expression levels correlate with TNM stage, lymph node metastasis, and post-treatment recurrence [70,71]. Similarly, circRNA_001275 (a microarray-derived identifier functionally validated via the miR-370-3p/Wnt7a axis) upregulated in cisplatin-resistant models and sponges miR-370-3p to enhance Wnt7a transcription, which activates the Wnt7a-LRP6-β-catenin cascade to establish a formidable survival barrier [72].

Resistance mechanisms can also be drug-specific; for instance, circPVT1 (circBase ID: hsa_circ_0001821) orchestrates 5-fluorouracil (5-FU) resistance by balancing FZD3-mediated Wnt activation with the suppression of ferroptosis via the upregulation of GPX4 and the downregulation of ACSL4. Its knockdown reduces IC50 by 67%, decreases multidrug resistance (MDR) proteins such as P-gp and MRP1, and restores ferroptosis sensitivity [73].

The therapeuic potential of circRNAs in overcoming chemotherapy resistance remains promising, offering novel avenues to improve patient prognosis. In contrast to oncogenic circRNAs, several tumor-suppressive circRNAs are downregulated in ESCC, where their absence facilitates tumor progression by failing to restrain Wnt signaling. For instance circ-ITCH (circBase ID: hsa_circ_0001141) is significantly depieted in ESCC, and its exogenous overexpression has been showen to inhibit proliferation and tumorigenicity. Mechanistically, hsa_circ_0001141 acts as a molecular sponge for miR-7, miR-17, and miR-214, effectively upregulating the E3 ubiquitin ligase ITCH [74]. The ITCH recognizes phosphorylated Dishevelled-2 (p-Dvl2) and mediates its K48-linked ubiquitination and subsequent degradation This action effectively blocks signal transmission from the receptor complex to β-catenin consequently suppressing Wnt/β-catenin activity [75]. The disruption of this hsa_circ_0001141/ITCH/p-Dvl2 axis represents a critical mechanism underlying sustained Wnt activation in ESCC, suggesting that hsa_circ_0001141 mimics or enhanced miRNA sponge strategies may represent innovative therapeutic directions.

In summary, circRNAs serve as structurally stable and functionally versatile modulators of the Wnt/β-catenin signaling pathway in ESCC, primarily operating through miRNA sponging mechanism. By orchestrating this central axis, these molecules drive malignant progression, promote EMT, and facilitate resistance to both radiotherapy and chemotherapy. While their diagnostic and therapeutic potential is considerable, achieving successful clinical translation requires overcoming significant hurdles related to functional complexity and delivery efficiency. Consequently, future research must prioritize the elucidation of these expansive regulatory networks to develop precise, mechanism-based intervention strategies. Given their exceptional stability and diverse regulatory roles, circRNAs represent promising candidates for clinical biomarker development, particularly for predicting therapeutic response and managing radio- and chemoresistance in ESCC.

Notably, the regulatory landscape of ncRNAs in ESCC is further defined by the miR-455-3p/Wnt/β-catenin axis. This specific miRNA exerts a dual regulatory role by orchestrating both malignant progression and chemoresistance. By concurrently suppressing negative regulators of the Wnt and TGF-β pathways, miR-455-3p expands the subpopulation of tumor-initiating cells (T-ICs), which directly drives tumor aggressiveness while conferring resistance to cisplatin and 5-FU. Furthermore, this landscape is increasingly complexified by epitranscriptomic modifications, such as the N^6^-methyladenosine (m^6^A). Emerging evidence suggests that m^6^A ‘writers’ like METTL3 and ‘erasers’ like ALKBH5 play complementary roles in modulating the stability of oncogenic transcripts, providing an additional layer of control over the therapeutic resistance and T-IC expansion observed in ESCC [76]. This specific miRNA exerts a dual regulatory role by orchestrating both malignant progression and chemoresistance. By concurrently suppressing negative regulators of the Wnt and TGF-β pathways, miR-455-3p expands the subpopulation of tumor-initiating cells (T-ICs), which directly drives tumor aggressiveness while conferring resistance to cisplatin [77]. These findings underscore that nodal regulators, namely miR-1275 and miR-455-3p, synchronize stemness maintenance with therapeutic failure through the Wnt pathway. Representative ncRNAs modulate chemoradiotherapy resistance and stemness are summarized in Table 3.

**Table 3:** Modulation of Chemoradiotherapy Resistance and Stemness.

Category	ncRNA/Axis	Expression in ESCC	Biological Effects	Clinical Relevance	Potential Therapeutic	Experimental Validation Level	Reference
miRNA	miR-942/sFRP4-GSK3β-TLE1/Wnt/β-catenin	Upregulated	Promotes cancer stem-like traits and tumorigenesis	poor prognosis	miR-942 inhibition	Clinical + *in vitro* + *in vivo*	[61]
miRNA	miR-1275/3′-UTR/WNT1/Wnt/β-catenin	Downregulated	Inhibits radioresistance, EMT, and malignant progression	Predictor of radioresistance	miR-1275 restoration	*In vitro*	[63]
miRNA	miR-301a/WNT1	Downregulated	Suppresses radioresistance, proliferation, EMT and malignant progression	Indicate treatment failure and radioresistance phenotype	miR-301a mimics	*In vitro*	[64]
circRNA	hsa_circRNA_0014879/miR-217/Wnt/β-catenin	Upregulated	Promotes radioresistance, proliferation, EMT, migration and invasion	Radioresistance and survival	hsa_circRNA_0014879 knockdown; miR-217 restoration; Wnt3 inhibition	Clinical + *in vitro* + *in vivo*	[65]
circRNA	hsa_circ_0000277/miR-873-5p/β-catenin, Bcl-2, Survivin	Upregulated	Promotes cisplatin resistance, proliferation and migration	Poor prognosis; TNM stage	has_circ_0000277 knockdown; miR-873-5p restoration; SOX4 inhibition	Clinical + *in vitro* + *in vivo*	[70]
circRNA	circRNA_001275/miR-370-3p/Wnt7a-LRP6-β-catenin	Upregulated (cisplatin-resistant cells)	Enhances cisplatin resistance	Associated with chemoresistance	Targeting circRNA_001275	Clinical + *in vitro*	[72]
circRNA	hsa_circ_0001821/miR-30a-5p/FZD3/β-catenin/c-Myc; GPX4/ACSL4	Upregulated (5-FU-resistant cells)	Promotes chemoresistance; suppresses ferroptosis	Associated with chemoresistance	hsa_circ_0001821 knockdown	Clinical + *in vitro* + *in vivo*	[73]
circRNA	hsa_circ_0001141/miR-7/17/214/ITCH/Dishevelled-2 (p-Dvl2)/Wnt/β-catenin	Downregulated	Suppresses proliferation and tumorigenesis	Potential biomarker and tumor suppressor	hsa_circ_0001141 restoration signaling	Clinical + *in vitro* + *in vivo*	[74]

### 3.4 The Complexity of Cross-Talk: ceRNA Networks

The regulatory landscape of ESCC is characterized by interconnected ceRNA networks rather than isolated, linear pathways. Within this framework, lncRNAs and circRNAs act as molecular sponges, sequestering specific miRNAs to prevent the degradation of Wnt-related transcripts. The DDX11-AS1/miR-30d-5p/SNAI1 axis serves as a quintessential example of this multi-layered control, coordinating EMT and β-catenin activity. While such cross-talk ensures precise gene regulation, it simultaneously introduces significant therapeutic complexity. Targeting a single ncRNA node may trigger unintended compensatory adjustments within the broader network, thereby diminishing the sustained efficacy of targeted interventions. Future investigations must transition from isolated, single-axis studies toward an integrated systems biology approach to fully resolve these complex interaction maps.

## 4 Therapeutic Targeting of ncRNAs: Pharmacological Strategies and Translational Perspectives

While the aforementioned mechanisms underscore the essential role of ncRNAs in regulating Wnt/β-catenin signaling in ESCC, translating these molecular insights into clinical practice necessitates a comprehensive understanding of ncRNA-mediated resistance landscapes. Such insights are pivotal for developing personalized therapeutic regimens tailored to individual patient profiles [60]. Furthermore, recent breakthroughs in nucleic acid chemistry and sophisticated drug delivery platforms—such as nanoparticle-based systems—have facilitated the development of targeted pharmacological interventions specifically designed to modulate dysregulated ncRNA nodes in ESCC.

### 4.1 RNA-Based Therapeutics: ASOs (Antisense Oligonucleotides), Antagomirs, and Mimics

Oncogenic miRNAs (oncomiRs) incorporating miR-141 and miR-942 [21,61], activate Wnt/β-catenin signaling by inhibiting endogenous negative regulators, representing prime targets for antagomirs. Chemically modified antisense oligonucleotides complementary to these miRNAs can restore the expression of SOX17, ZNRF3 and the sFRP4/GSK-3β/TLE1 axis, thereby reinstating inhibitory control over the Wnt pathway. By contrast, tumor-suppressive miRNAs as miR-301a and miR-1275 [63,64], which are typically downregulated in radioresistant ESCC, can be delivered as synthetic miRNA mimics or agomirs to silence WNT1 expression, consequently sensitizing cells to radiosensitive.

For lncRNAs, antisense oligonucleotide (ASO)-mediated silencing offers a direct approach to inhibiting oncogenic transcripts. Preclinical studies suggest that ASOs targeting CCAT2 could suppress β-catenin/WISP1 signaling and metastatic dissemination [49]. Similarly, ASOs directed against DDX11-AS1 might disrupt the miR-30d-5p/SNAI1/ZEB2 regulatory axis, preventing EMT and the nuclear translocation of β-catenin [53]. Moreover, targeting PVT1 or SNHG16 with gapmers or siRNAs could attenuate Wnt pathway activation and enhance chemosensitivity [47,55]. Conversely, replacement therapies using plasmid vectors or modified oligonucleotides to restore the expression of tumor-suppressive lncRNAs—such as lncRNA-NEF [56], LINC00675 [57], and GASL1 [58]—represent complementary strategies to inhibit aberrant Wnt signaling.

Circular RNAs, characterized by their exceptional stability and resistance to nuclease degradation, present unique therapeutic opportunities. For oncogenic circRNAs like hsa_circ_0001707 [39], hsa_circRNA_0014879 [65], and hsa_circ_0000277 [70,71], siRNAs or shRNAs targeting specific back-splice junctions can disrupt their sponge functions and restore miRNA-mediated suppression of Wnt targets. Furthermore, delivery of hsa_circ_0001141 mimics could enhance ITCH-mediated DVL2 degradation [74], thereby inhibiting signal transmission to β-catenin.

### 4.2 Drug Repurposing and Combination Strategies

Integrating ncRNA-targeting agents with existing Wnt pathway inhibitors may synergistically overcome compensatory resistance mechanisms. For instance, combining miR-301a or miR-1275 mimics with radiotherapy could leverage their roles in modulating radioresistance by specifically targeting WNT1 [63,64]. In chemotherapy-resistant ESCC manifested through circRNA-mediated Wnt activation (e.g., circRNA_001275 [72] or hsa_circ_0001821 axis [73]), combining cisplatin or 5-fluorouracil with siRNAs targeting these circRNAs may reverse resistance by restoring ferroptosis sensitivity and inhibiting Wnt/β-catenin signaling. Additionally, computational drug repurposing can be facilitated through using publicly available databases like ncRNADrug and ChEMBL, which catalog complex drug-ncRNA interactions [78,79]. Systematically analyzing these resources may identify FDA-approved compounds ranging from chemotherapy agents to specific kinase inhibitors—that modulate ncRNAs represented by CCAT2 or has_circ_0001707, Such bioinformatics approaches provide immediate, actionable candidates for combination clinical trials in ESCC.

### 4.3 Delivery Systems and Clinical Considerations

While preclinical models have demonstrated the potential therapeutic value of targeting the ncRNA-Wnt axis, these strategies currently remain in the early research stages. Critical challenges persist, including the insufficient biostability of RNA-based agents against nuclease-mediated degradation, suboptimal delivery efficiency into the dense and complex esophageal tumor microenvironment, and a current lack of tissue-specific targeting mechanisms. Furthermore, unintended off-target effects and potential systemic immunogenicity—stemming from the pleiotropic nature of ncRNAs, which often modulates multiple biological pathways beyond the Wnt axis—necessitate a stringent evaluation of their safety profiles in humans. Future efforts should prioritize the development of tumor-specific vehicles, such as lipid nanoparticles (LNPs) or ligand-functionalized exosomes, to enhance specificity and reduce systemic toxicity. Such advancements leverage the innate stability of circRNAs and the inherent targeting capabilities of exosomal membranes, offering a promising roadmap for the generation of ESCC therapeutics [59,80].

### 4.4 Clinical Validation and Emerging Biomakers

While ncRNA-based therapeutics for ESCC are predominantly in the preclinical stage, their role as validated biomarkers in patient cohorts has gained significant traction. Genomic analyses, like those from the TCGA-ESCC cohort, have already confirmed the clinical relevance of Wnt pathway dysregulation in predicting patient prognosis.

Diagnostic and Prognostic Signatures: Several ncRNAs have been validated in clinical specimens as reliable indicators of disease progression. For instance, miR-141 has been identified as a potential early detection marker, particularly in patients with a history of alcohol consumption. Similarly, the downregulation of miR-33a-5p and miR-30a-3p in patient tissues strongly correlates with advanced TNM staging and poor 5-year overall survival (OS) [31,37,41].

Liquid Biopsy Potential: The detection of ncRNAs in biological fluids offers a non-invasive approach to clinical monitoring. Notably, miR-10527-5p levels are significantly depleted in the plasma exosomes of ESCC patients with lymph node metastasis, serving as a validated diagnostic indicator [45]. Furthermore, circular RNAs like hsa_circ_0001707 and hsa_circ_0000277 have demonstrated high sensitivity as diagnostic biomarkers in clinical cohorts, with their expression levels closely linked to post-treatment recurrence [65,71].

Predictive Value for Therapy: Beyond diagnosis, ncRNA profiles are emerging as tools for patient stratification. Low expression of miR-301a or miR-1275 in patient biopsies is a validated predictor of radioresistance and treatment failure, providing a potential molecular basis for adjusting radiotherapy regimens [39,75].

Future clinical investigations should prioritize as follows: Firstly, synergistic combination regimens pairing ncRNA modulators with conventional chemotherapy or radiotherapy to overcome acquired resistance; Then, biomarker-driven patient stratification based on ncRNA expression profiles (e.g., high CCAT2 or hsa_circ_0001707 expression as indicators for ASO therapy); and develop organ-specific and tumor-targeted delivery systems to enhance therapeutic efficacy while minimize systemic toxicity. By elucidating the complexity of ncRNA-mediated Wnt signaling networks and developing innovative pharmacological interventions, ncRNA-targeted therapies offer substantial promise for improving therapeutic outcomes in ESCC.

## 5 Conclusions and Perspectives

A multiple of signaling cascades contribute to the development of ESCC, among which the Wnt/β-catenin pathway plays a central role in driving invasion, metastasis, and therapeutic resistance. Accumulating evidence indicates that various ncRNA species—miRNAs, lncRNAs, and circRNAs—regulate the activity of this signaling axis through diverse mechanisms, thereby shaping ESCC biological behavior.

Oncogenic miRNAs (e.g., miR-141, miR-106b-3p) and lncRNAs (e.g., CCAT2, PVT1) aberrantly activate Wnt/β-catenin signaling by targeting endogenous suppressors or relieving inhibitory constraints, thus promoting proliferation, migration, and invasion. Conversely, tumor-suppressive miRNAs (e.g., miR-301a, miR-1275) and lncRNAs (e.g., lncRNA-NEF, LINC00675) restrain pathway activation, consequently reducing metastatic potential. Oncogenic circRNAs (e.g., has_circ_0001707, hsa_circRNA_0014879) function as molecular sponges to sequester miRNAs and alleviate the repression of Wnt signaling, whereas tumor-suppressive circRNAs like hsa_circ_0001141 inhibit progression by upregulating pathway inhibitors. Together, these ncRNAs form a multilayered regulatory network that orchestrates ESCC malignancy in preclinical models.

Despite these mechanistic insights, the precise regulatory logic by which Wnt/β-catenin signaling contributes to ESCC remains incompletely defined. Existing studies rely heavily on *in vitro* cell lines and animal models, which provide essential mechanistic insights, they carry notable limitations, regarding biological heterogeneity. ESCC is characterized by significant molecular and anatomical diversity among patients, and standardized cell lines may fail to capture the complex genomic landscape found in actual clinical cohorts. This heterogeneity complicates the establishment of universal ncRNA signatures for risk stratification. 

In particular, there is a lack of systematic, large-scale clinical validation in specimens—especially longitudinal samples collected across different treatment stages—to track dynamic alterations in pathway activity. Evidence from clinical cohorts and advanced models, including patient-derived organoids (PDOs) and patient-derived xenografts (PDX), remain limited. From a pathological standpoint, the dysregulation of ncRNA-mediated Wnt/β-catenin signaling appears to be a recurrent molecular hallmark associated with ESCC progression and therapeutic resistance failure.

Future research should prioritize the integration of multi-omics approaches and functional studies to clarify: (1) the molecular basis of crosstalk between Wnt/β-catenin axis and other core pathways, and its integrated role in malignant progression; (2) how ncRNAs, particularly circRNAs and lncRNAs, function as nodal regulators of these interaction networks. Importantly, while these mechanistic insights offer experimental promise.

Translating these experimental insights into clinically validated applications remains in its infancy. Success depends on addressing fundamental hurdles, including molecular stability and tissue-specific delivery. The development of organ-specific delivery systems is essential to overcome enzymatic degradation in circulation and ensure specific accumulation in esophageal malignancies while minimizing systemic exposure. Conversely, although replacement therapies using synthetic mimics or ASOs offer potential value, they are currently hindered by the risk of unintended off-target interactions. Because a single ncRNA can modulate hundreds of target genes, its therapeutic modulation may unintentionally disrupt essential physiological pathways, potentially leading to systemic toxicity.

The utilization of publicly databases, including ncRNADrug, ChEMBL, and PubChem BioAssay, will be instrumental in identifying small molecules with the potential to modulate ncRNA expression or function, thereby contributed to expediting drug discovery. However, the clinical translation of these RNA-based therapeutics critically depends on the development of efficient delivery systems, including chemically modified oligonucleotides with enhanced nuclease resistance and tumor-targeted nanoparticle formulations, to ensure specific accumulation in esophageal malignancies while minimizing systemic toxicity.

Given that single-target Wnt inhibition may be undermined by pathway compensation, exploring combination strategies is a critical future direction. This includes rational scheduling of Wnt inhibitors with chemotherapy or radiotherapy, combining Wnt targeting with inhibitors of interacting pathways such as PI3K/Akt; the integration of ncRNA-modulating agents (e.g., ASOs against CCAT2 or SNHG16, miR-301a mimics) with conventional therapeutics to overcome chemoradioresistance; and evaluating the potential of combining Wnt-modulating traditional Chinese medicine-derived compounds. Particular attention should be directed towards circRNA-based interventions, where siRNAs targeting oncogenic circRNAs (e.g., hsa_circ_0001707, hsa_circ_0000277) or hsa_circ_0001141 mimics may offer novel avenues to modulate Wnt signaling with enhanced stability and specificity. 

In conclusion, by rigorously resolving the complexity of ncRNA-Wnt signaling networks and refining delivery technologies, these strategies may eventually complement conventional therapies. However, sustained clinical validation in robust patient is essential before these interventions can meaningfully improve survival outcomes for ESCC patients.

## Data Availability

Not applicable.
